# Supplementary material for: rRNA expansion segments mediate ribosome dimerization as a conserved stress response
Source: Nucleic Acids Res. 2026 Apr 20;54(7):gkag354. doi: 10.1093/nar/gkag354 (PMC13092973; doi:10.1093/nar/gkag354)
Supplement: gkag354_Supplemental_Files [file gkag354_supplemental_files.zip › NAR_20260307_SI.pdf]

rRNA Expansion Segments Mediate Ribosome Dimerization as a Conserved Stress Response

Wenhong Jiang[1,2,#], Chen Chen[1,3,#], Xing Wang[1,#], Wei Huang[4], Dawid Krokowski[5,6], Ziyao Chen[1], Jiahao Xie[7], Zhaoming Su[7], Maria Hatzoglou[5,*], and Derek J Taylor[4,8,*], Qiang Guo[1,3,*]

1. State Key Laboratory of Membrane Biology, Peking-Tsinghua Center for Life Sciences, School of Life Sciences, Peking University, Beijing 100871, China
2. Key Laboratory of Fermentation Engineering (Ministry of Education), National 111 Center for Cellular Regulation and Molecular Pharmaceutics, Hubei University of Technology, Wuhan, Hubei, 430068, China
3. Changping Laboratory, Beijing 102206, China
4. Department of Pharmacology, Case Western Reserve University, Cleveland, OH 44106, USA
5. Department of Genetics and Genome Sciences, Case Western Reserve University, Cleveland, OH 44106, USA
6. Department of Molecular Biology, Institute of Biological Sciences, Maria Curie-Skłodowska University, Lublin, Poland
7. The State Key Laboratory of Biotherapy, West China Hospital, Sichuan University, Chengdu, China
8. Department of Biochemistry, Case Western Reserve University, Cleveland, OH 44106, USA

#. These authors contributed equally
*. Corresponding authors: mxh8@case.edu (M.H.), djt36@case.edu (D.T.) and guo.qiang@pku.edu.cn (Q.G.)

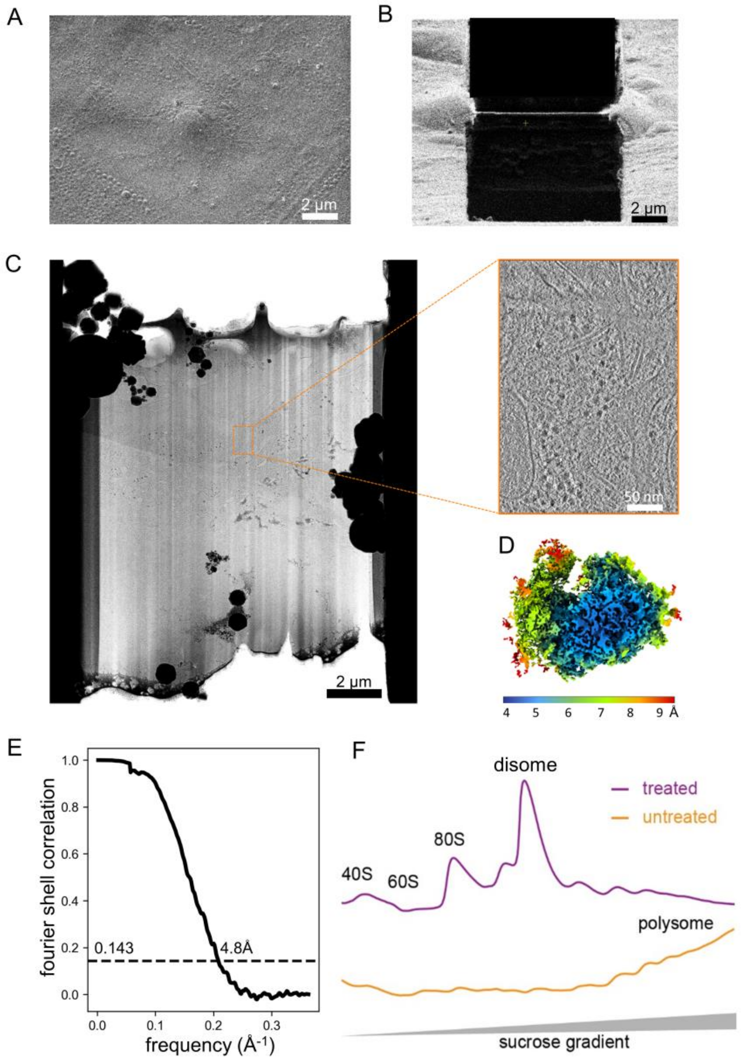

Figure S1. Experimental pipeline from sample preparation to structural analysis.
(A) Cryo-SEM image showing neuronal cells cultured on EM grids.
(B-C) Cellular lamellae with a thickness of <200 nm prepared using cryo-focused ion beam milling. The FIB image (B) and the corresponding TEM image (C) are shown. The boxed region indicates the area targeted for tilt-series acquisition, with tomographic slices shown.
(D) The subtomogram averaging map of the ribosome, color-coded according to local resolution.
(E) Fourier shell correlation (FSC) curve used to assess the overall resolution of the reconstructed map. The resolution was estimated using the FSC = 0.143 criterion.
(F) Polysome profiling using sucrose gradients, comparing control (orange) and puromycin-treated (purple) neuronal cell samples.
Scale bars: 2 µm in (A) (B) and left of (C), 50 nm in right of (C).

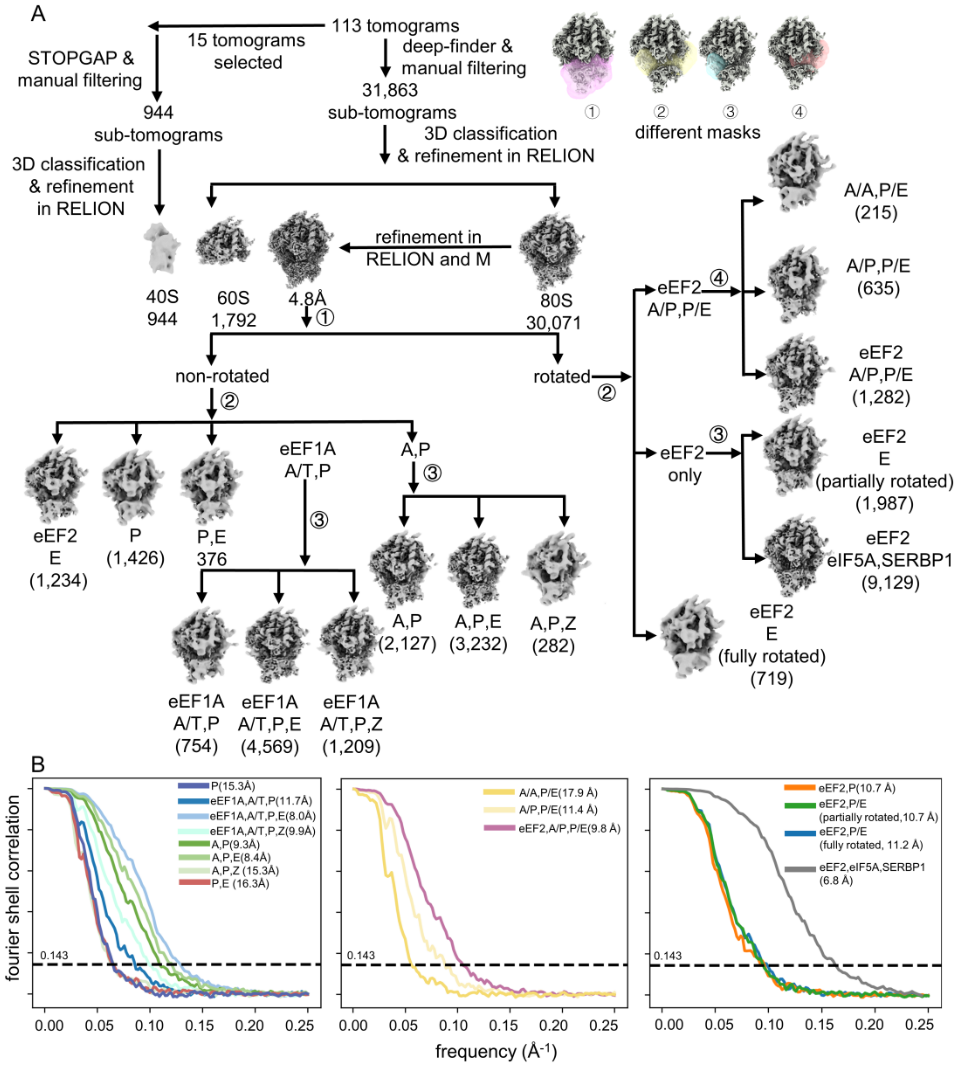

Figure S2. Ribosome classification pipeline for cryo-ET datasets.
(A) Diagram of the cryo-ET data analysis workflow. Four region-specific masks applied at different stages of the classification are shown in the top right (pink: small subunit, yellow: A/P/E sites and GTPase Activation Center, cyan: E/Z tRNA sites, pink: GTPase Activation Center). The final particle numbers used for each reconstructed map are indicated.
(B) Fourier shell correlation (FSC) curves for the subtomogram averages corresponding to the different classified ribosome conformations. The resolution was provided for each map (FSC = 0.143).

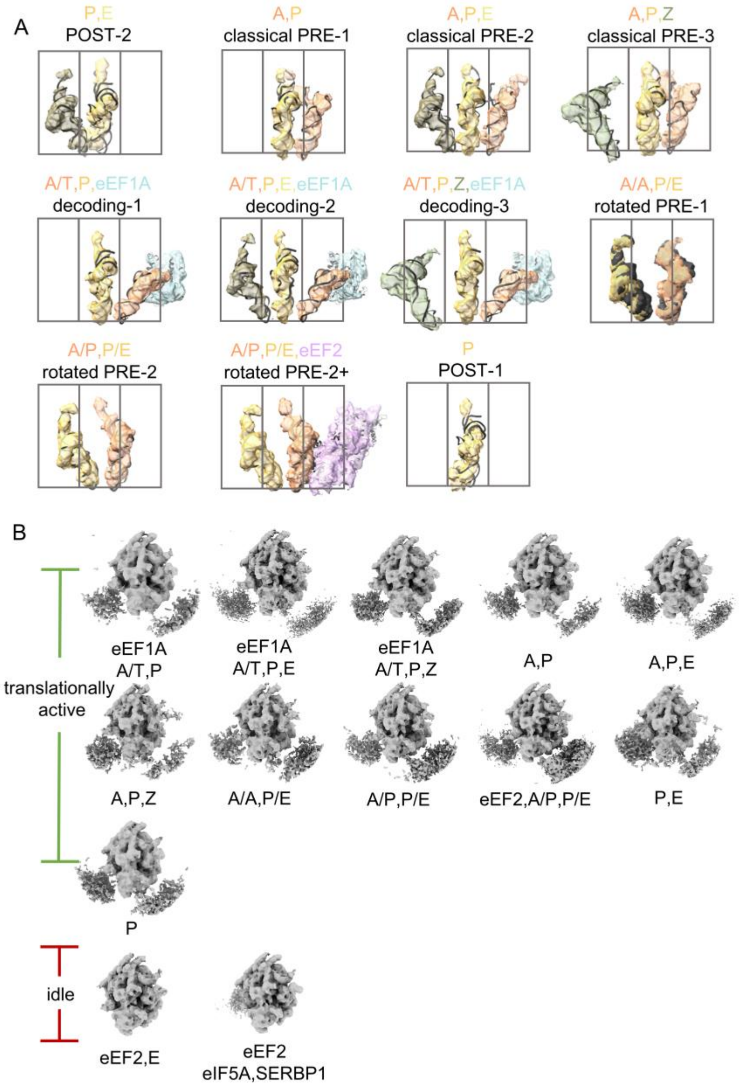

Figure S3. The assigned translation states of ribosomes.

(A) Comparison of the positions of tRNAs and translation elongation factors in each classified ribosome state. Densities corresponding to tRNAs (pink, orange, and yellow), eEF1A (cyan), and eEF2 (purple) are segmented and fitted with previously determined atomic models using rigid-body docking. Grey frames indicate the relative positions of the E, P, and A sites (from left to right).

(B) The neighborhood density of each ribosome translation states. Particles corresponding to each state were reconstructed using a larger box size without masking to visualize neighboring ribosomes within the local cellular environment.

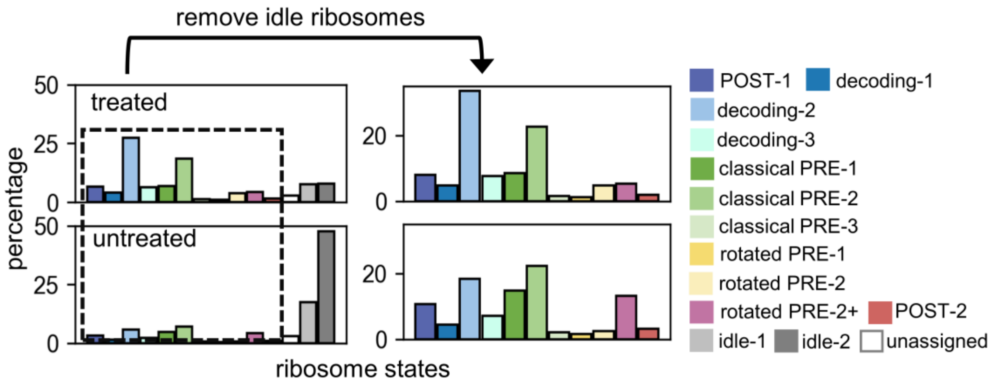

Figure S4. Comparative analysis of ribosome translation states populations.
Bar charts showing the translation states distribution of ribosome in puromycin-treated (upper) and untreated control (lower) samples. The left panels display the total ribosome population including all identified states, whereas the right panels show the relative abundance of each translation state after excluding idle ones (idle-1 and idle-2). Translation states are color-coded as indicated.

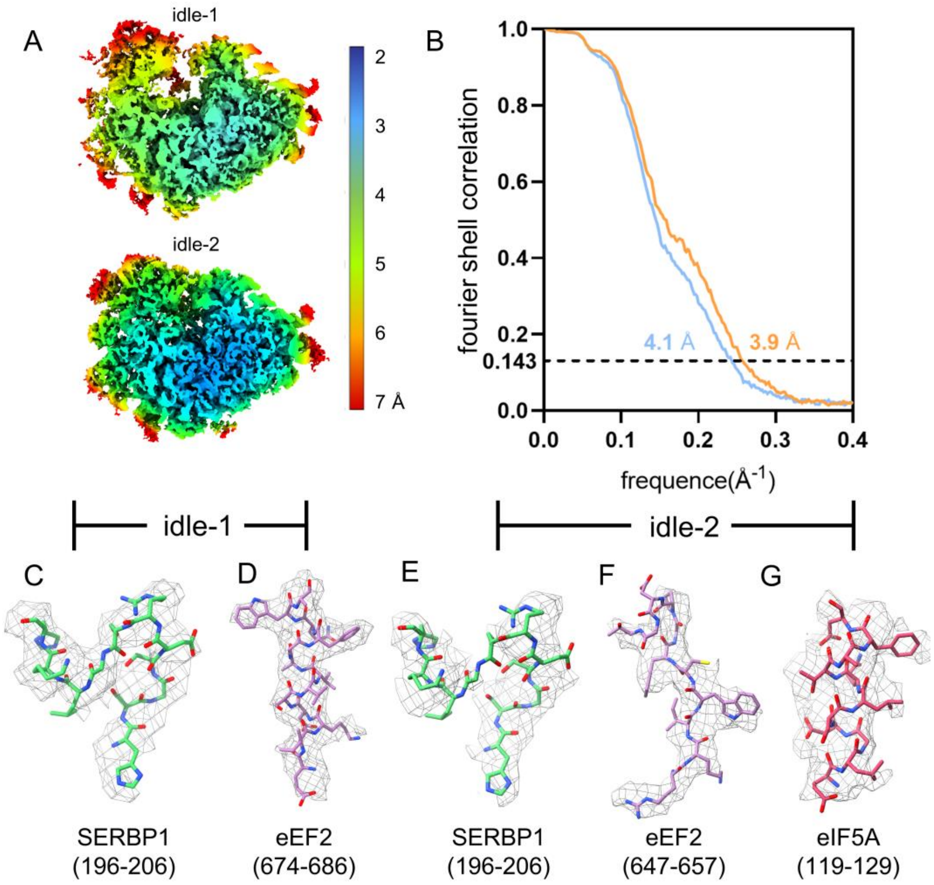

Figure S5. Cryo-EM analysis of idle ribosomes using GisSPA.
(A) Density maps of the two idle states, color-coded according to local resolution.
(B) Fourier shell correlation (FSC) curve used to assess the overall resolution of the reconstructed maps. The resolutions were estimated using the FSC = 0.143 criterion.
(C-D) Atomic model validation of the idle-1 ribosome showing density fitting for SERBP1 and eEF2 in representative regions.
(E-G) Atomic model validation of the idle-2 ribosome showing density fitting for SERBP1, eEF2 and eIF5A in representative regions.

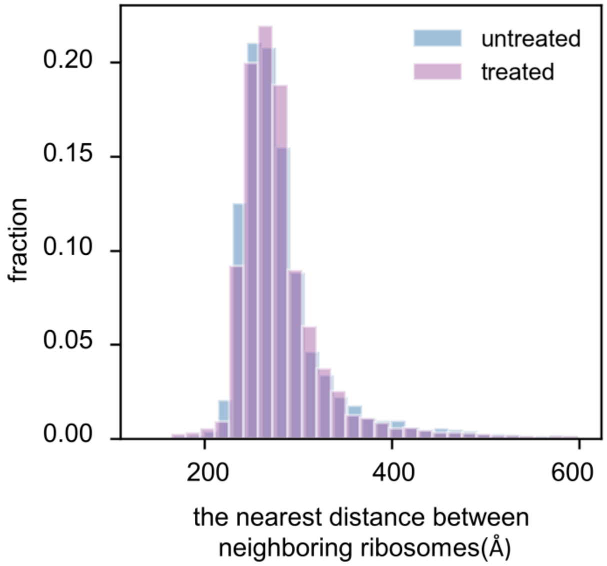

Figure S6. Distribution of nearest-neighbor distances between ribosomes.
For each ribosome, the spatial distance to its nearest neighboring ribosome was calculated, and the distribution of nearest-neighbor distances was plotted. Puromycin-treated samples are shown in purple, and untreated samples in blue. The x-axis represents the nearest-neighbor distance (Å), and the y-axis indicates the fraction of ribosome pairs.

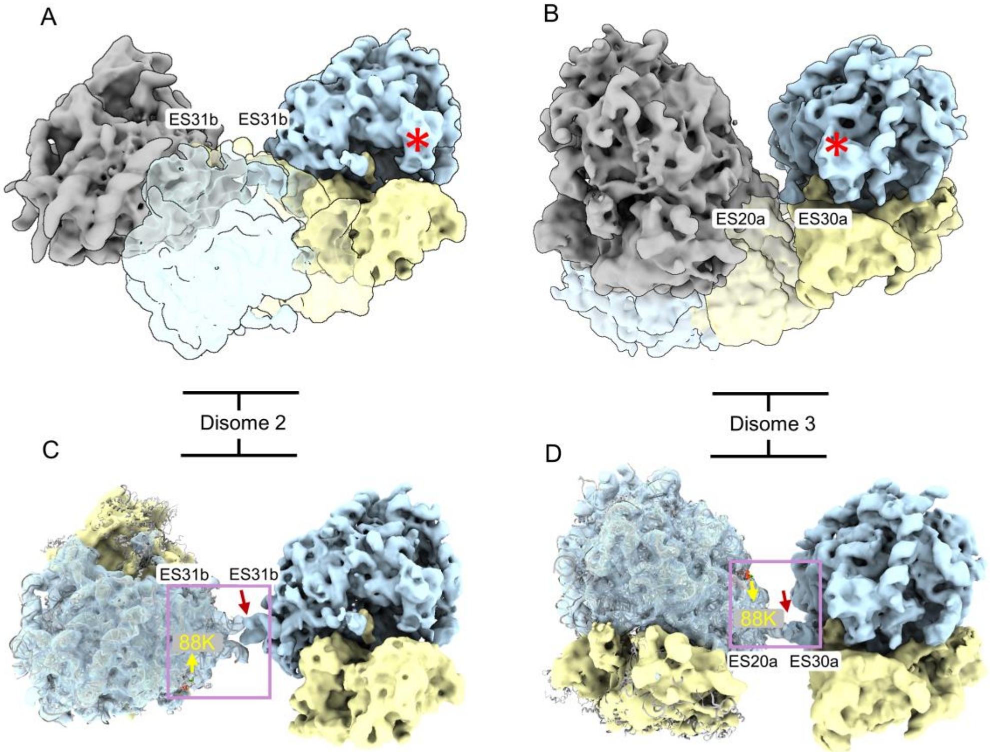

Figure S7. Structural characteristics of the two hibernating disomes.
(A-B) Structural comparison of Disome 2 (A) and Disome 3 (B) identified in this study with a previously reported collided–stalled disome (EMDB-4427). Disomes identified in this work are shown in gray and aligned to the collided–stalled disome using the ribosome marked with "*" as the reference. In the collided–stalled disome, the large and small ribosomal subunits are colored blue and yellow, respectively.
(C-D) Spatial relationship between the ribophagy-associated ubiquitination site (Lys88, indicated by a yellow arrow) in ribosomal protein uL23 (red) (1) and the dimer interface identified in this study (red arrow).

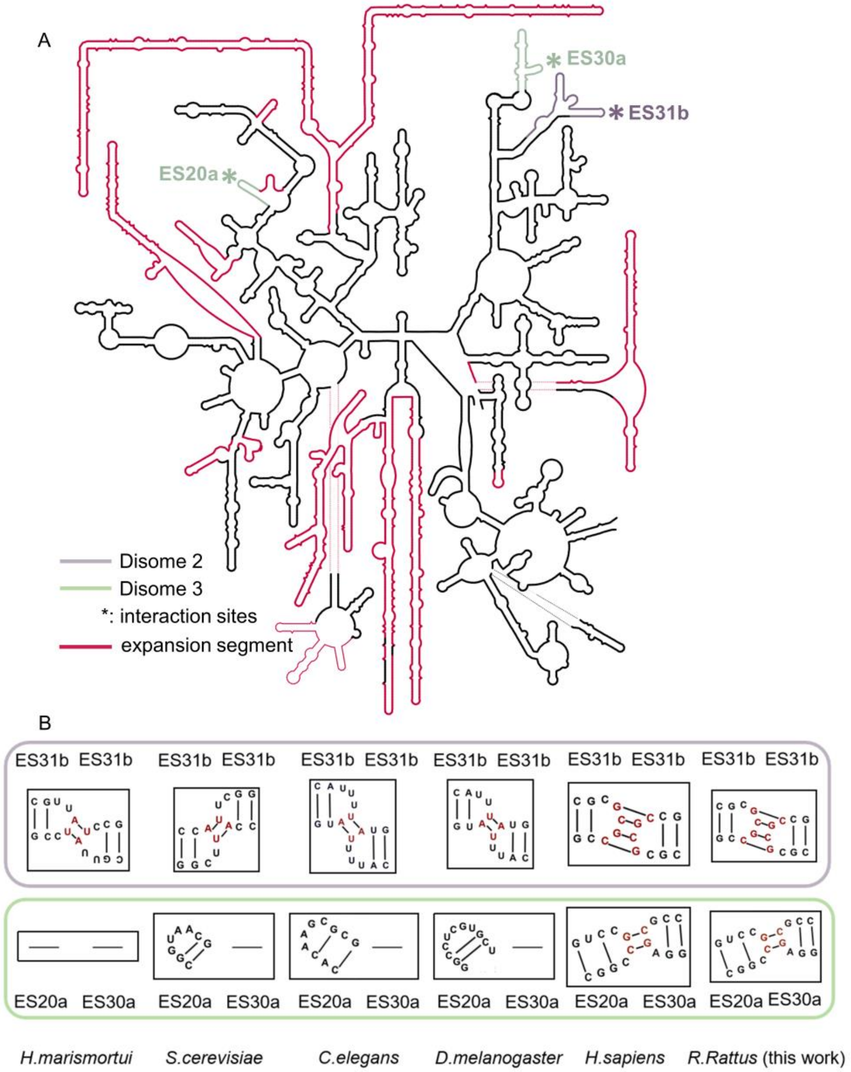

Figure S8. The rRNA sequence analysis at disome interfaces.

(A) The secondary structure of rat 28S rRNA is illustrated, with expansion segments highlighted in red. The interface regions associated with Disome 2 (labeled in purple) and Disome 3 (labeled in green) are denoted by asterisks.

(B) The inferred base-pairings within interface regions of Disome 2 (purple boxed) and Disome 3 (green boxed) across different species. Noting in some species, specific expansion segments are absent and are denoted with ‘-‘.

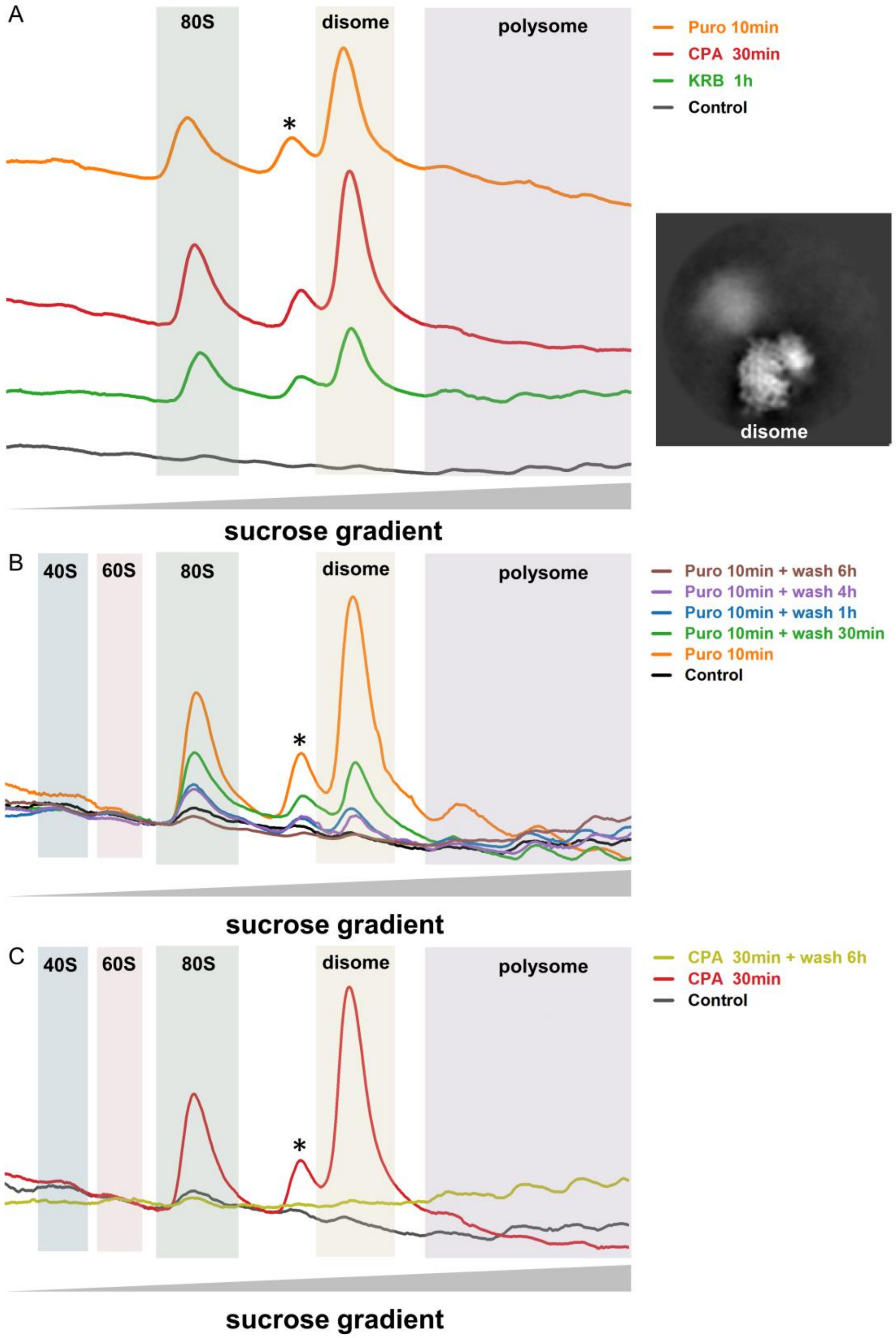

Figure S9. Disome formation in response to different stress stimuli in C6 glioma cells is reversible.

(A) Polysome profiling shows that ribosome disomes increase in response to puromycin administration, CPA administration, or amino acid starvation (KRB media). The right panel shows the 2D projection of the purified disomes, as determined by the single-particle cryo-EM.

(B) Ribosome disomes formed under puromycin administration is reversible following puromycin removal. Polysome profiling results were shown under conditions of puromycin treatment for 10 min, as well as at 30 min, 1 h, 4 h, and 6 h after removal of puromycin following the 10-min treatment.
(C) Ribosome disomes formed under CPA administration is reversible following CPA removal. Polysome profiling results were shown under conditions of CPA treatment for 30 min, as well as at 6 h after removal of puromycin following the 30-min treatment.
The * peak is indicative of a putative 60S-80S disome.

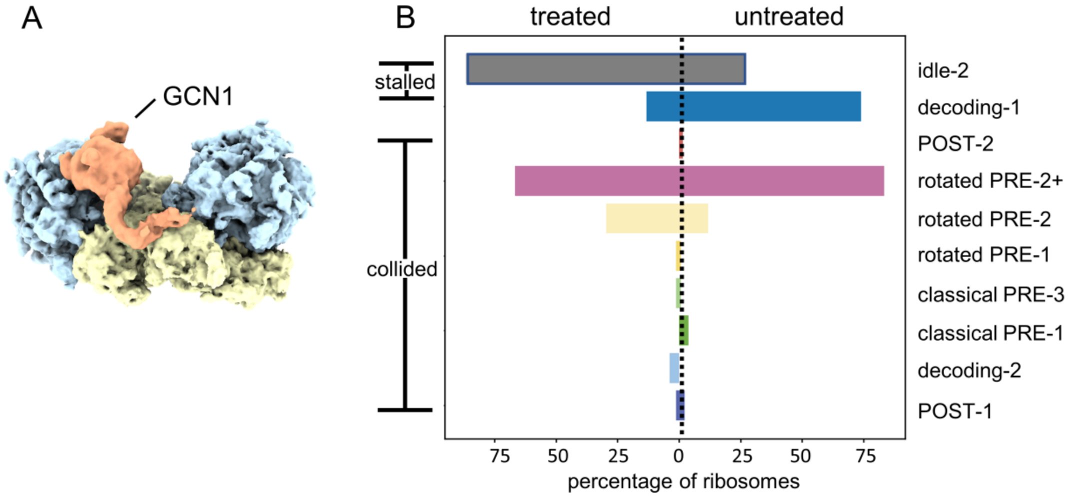

Figure S10. In situ analysis of ribosome collision.
(A) In situ subtomogram average of GCN1-bound (orange) collided-stalled disomes.
(B) The ribosome translation states distribution of stalled or collided ribosomes in the untreated and puromycin-treated group. Bar charts showing the abundance of ribosome translation states in puromycin-treated (left) and control (right) samples of stalled and collided ribosome, respectively.

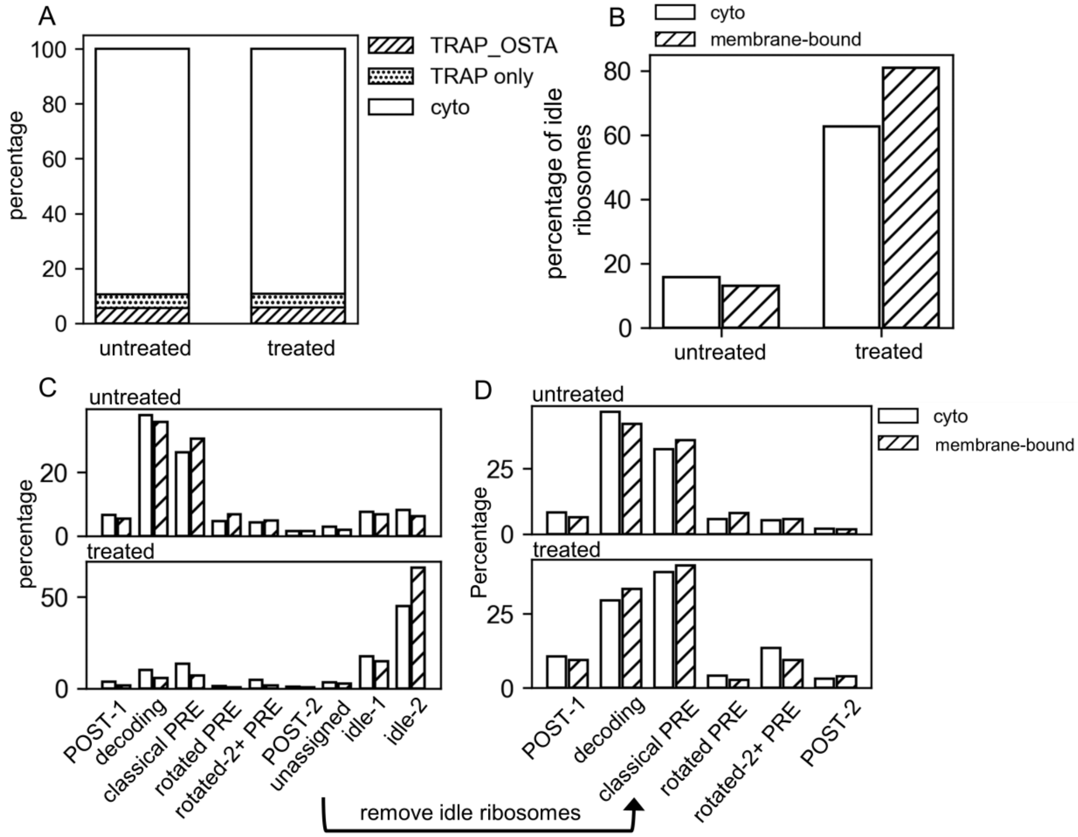

Figure S11. Comparison analysis of cytoplasmic and membrane-bound ribosomes.
(A) The abundance of cytoplasmic ribosomes, membrane-bound ribosome with/without OSTA in untreated (left) and puromycin-treated group (right).
(B) The population of idle ribosomes (idle-1 and idle-2) in the cytoplasm (white bars) or bound to the membrane (hatched bars) in untreated (left) and puromycin-treated (right) group.
(C-D) The translation states distribution of ribosomes in untreated (upper) and puromycin-treated (lower) group with (C) and without (D) the inclusion of idle ones (idle-1 and idle-2). The data was divided into two groups: ribosomes in the cytoplasm (white bars) and membrane-bound ribosomes (hatched bars).

Table S1. Single particle cryo-EM and cryo-ET data collection.

Dataset	cryo-ET	cryo-ET	cryo-ET	cryo-ET	cryo-ET	cryo-ET	cryo-ET	cryo-ET	cryo-ET	cryo-ET	cryo-ET	cryo-ET	cryo-ET	SPA	SPA
State	decoding-1	decoding-2	decoding-3	classical PRE-1	classical PRE-2	classical PRE-3	rotated PRE-1	rotated PRE-2	rotated PRE-2+	POST-1	POST-2	idle-1	idle-2	idle-1	idle-2
EMDB	EMD-66239	EMD-66240	EMD-66241	EMD-66242	EMD-66243	EMD-66244	EMD-66250	EMD-66248	EMD-66249	EMD-66246	EMD-66245	EMD-66247	EMD-66251	EMD-66255	EMD-66256
**Data collection and processing**															
Microscope	Titan Krios G3	Titan Krios G3	Titan Krios G3	Titan Krios G3	Titan Krios G3	Titan Krios G3	Titan Krios G3	Titan Krios G3	Titan Krios G3	Titan Krios G3	Titan Krios G3	Titan Krios G3	Titan Krios G3	Titan Krios G4	Titan Krios G4
Magnification	64,000X	64,000X	64,000X	64,000X	64,000X	64,000X	64,000X	64,000X	64,000X	64,000X	64,000X	64,000X	64,000X	105,000X	105,000X
Voltage (kV)	300	300	300	300	300	300	300	300	300	300	300	300	300	300	300
Electron exposure (e-/Å²)	110	110	110	110	110	110	110	110	110	110	110	110	110	60	60
Defocus range (μm)	-2.0 to -5.0	-2.0 to -5.0	-2.0 to -5.0	-2.0 to -5.0	-2.0 to -5.0	-2.0 to -5.0	-2.0 to -5.0	-2.0 to -5.0	-2.0 to -5.0	-2.0 to -5.0	-2.0 to -5.0	-2.0 to -5.0	-2.0 to -5.0	-0.8 to -1.6	-0.8 to -1.6
Pixel size (Å)	2.74	2.74	2.74	2.74	2.74	2.74	2.74	2.74	2.74	2.74	2.74	2.74	2.74	1.21	1.21
Symmetry imposed	C1	C1	C1	C1	C1	C1	C1	C1	C1	C1	C1	C1	C1	C1	C1
Initial particls images (no.)	31,863	31,863	31,863	31,863	31,863	31,863	31,863	31,863	31,863	31,863	31,863	31,863	31,863	117,690	117,690
Final particle images (no.)	754	4,569	1,209	2,127	3,232	282	215	635	1,282	1,426	376	3,952	9,129	48,855	35,924
Map resolution (Å)	11.7	7.8	10.1	9.1	8.3	16.3	18.6	11.4	9.6	9.8	15	8.1	6.8	3.9	4.1
FSC threshold	0.143	0.143	0.143	0.143	0.143	0.143	0.143	0.143	0.143	0.143	0.143	0.143	0.143	0.143	0.143
Map sharpening B factor (Å²)	-100	-96	-100	-101	-101	-105	-109	-105	-98	-98	-103	-101	-122	-100	-100

Movie S1. The assigned translation states of ribosomes in this work.

Reference:

1. Ossareh-Nazari, B., Niño, C.A., Bengtson, M.H., Lee, J.W., Joazeiro, C.A. and Dargemont, C. (2014) Ubiquitylation by the Ltn1 E3 ligase protects 60S ribosomes from starvation-induced selective autophagy. *J Cell Biol*, **204**, 909-917.
